# MeDEStrand: an improved method to infer genome-wide absolute methylation levels from DNA enrichment data

**DOI:** 10.1186/s12859-018-2574-7

**Published:** 2018-12-22

**Authors:** Jingting Xu, Shimeng Liu, Ping Yin, Serdar Bulun, Yang Dai

**Affiliations:** 1 Department of Bioengineering, University of Illinois at Chicago, Chicago, IL USA; 2 Division of Reproductive Science in Medicine, Department of Obstetrics and Gynecology, Feinberg School of Medicine, Northwestern University, Chicago, IL USA

**Keywords:** DNA methylation, MeDIP-seq, RRBS, CpG bias, Sigmoid function

## Abstract

**Background:**

DNA methylation of CpG dinucleotides is an essential epigenetic modification that plays a key role in transcription. Widely used DNA enrichment-based methods offer high coverage for measuring methylated CpG dinucleotides, with the lowest cost per CpG covered genome-wide. However, these methods measure the DNA enrichment of methyl-CpG binding, and thus do not provide information on absolute methylation levels. Further, the enrichment is influenced by various confounding factors in addition to methylation status, for example, CpG density. Computational models that can accurately derive absolute methylation levels from DNA enrichment data are needed.

**Results:**

We developed “MeDEStrand,” a method that uses a sigmoid function to estimate and correct the CpG bias from enrichment results to infer absolute DNA methylation levels. Unlike previous methods, which estimate CpG bias based on reads mapped at the same genomic loci, MeDEStrand processes the reads for the positive and negative DNA strands separately. We compared the performance of MeDEStrand to that of three other state-of-the-art methods “MEDIPS,” “BayMeth,” and “QSEA” on four independent datasets generated using immortalized cell lines (GM12878 and K562) and human primary cells (foreskin fibroblasts and mammary epithelial cells). Based on the comparison of the inferred absolute methylation levels from MeDIP-seq data and the corresponding reduced-representation bisulfite sequencing data from each method, MeDEStrand showed the best performance at high resolution of 25, 50, and 100 base pairs.

**Conclusions:**

The MeDEStrand tool can be used to infer whole-genome absolute DNA methylation levels at the same cost of enrichment-based methods with adequate accuracy and resolution. R package MeDEStrand and its tutorial is freely available for download at https://github.com/jxu1234/MeDEStrand.git.

**Electronic supplementary material:**

The online version of this article (10.1186/s12859-018-2574-7) contains supplementary material, which is available to authorized users.

## Background

DNA methylation of CpG dinucleotides is an essential epigenetic modification that plays a key role in transcription regulation. Sequencing-based DNA methylation profiling techniques include whole-genome bisulfite sequencing (WGBS), reduced-representation bisulfite sequencing (RRBS), and enrichment-based methods, such as methylated DNA immunoprecipitation (IP) followed by high-throughput sequencing (MeDIP-seq) and methyl-CpG binding domain protein-enriched genome sequencing (MethylCap-seq/MBD-seq) [1, 2]. WGBS and RRBS are “gold standard” methods for DNA methylation studies [3, 4]. RRBS provides substantial coverage of CpGs in CpG islands but with lower CpG coverage genome-wide, while WGBS offers greater CpG coverage genome-wide but at a significantly higher cost. By converting unmethylated cytosine to uracil (displayed as thymine following PCR amplification), leaving methylated cytosine unconverted, the ratio of C-to-T conversion allows quantification of DNA methylation at single-base resolution on the scale from 0 to 1. MeDIP-seq [5] and MethylCap-seq/MBD-seq [6] are DNA enrichment-based methylation profiling methods. MeDIP-seq utilizes an anti-methylcytosine antibody to immunoprecipitate methylated single-stranded DNA fragments. MethylCap/MBD-seq utilizes the methyl-CpG binding domain of MBD family proteins to enrich for methylated double-stranded DNA fragments. The samples enriched for methylated DNA fragments can then be used to infer regional methylation status, providing the lowest cost per CpG covered genome-wide [1]. However, the absolute methylation levels in these enriched samples must be derived using computational models that eliminate the effects of confounding factors.

It has been shown that MeDIP-derived data need to be corrected for CpG density effects to obtain unbiased methylation levels [7, 8]. “MEDME” is one of the earliest methods developed to quantify the CpG density effect based on microarray-derived MeDIP-ChIP enrichment data from normal human melanocytes [8]. Acknowledging that methylation level and density of CpGs (within the enriched DNA fragments) are the main factors that affect the enrichment results, MEDME generates a fully methylated control sample to establish a one-to-one relationship between MeDIP-ChIP enrichment signals and the corresponding CpG density. The enrichment signal is explained by only one factor, i.e., the CpG density, which shows a sigmoidal relationship. A four-parameter logistic model is then used to fit the curve for CpG bias correction. However, MEDME-inferred absolute methylation level for a 1 kb window is at a low resolution.

“BATMAN” is another method for inferring CpG methylation levels from array-based data [7]. It assumes a linear CpG density effect and utilizes Bayesian inference to estimate the posterior distribution of the methylation parameters given the enrichment signals. BATMAN provides inferred absolute methylation values at high resolution of 50 or 100 bp. However, the process has been reported as time-consuming compared to other methods.

The “MEDIPS” method was subsequently developed for inferring CpG methylation levels from MeDIP-seq data based on a linear regression model [9, 10]. It assumes a linear CpG density effect for all regions and utilizes the information from the low CpG density region of the enrichment data itself to estimate the CpG density effect. MEDIPS has similar performance as BATMAN, but with significantly reduced running time.

Other methods have incorporated additional experimental data to make more accurate inferences of CpG methylation levels from DNA enrichment data. “BayMeth” is a Bayesian method that uses information from a fully methylated control sample [11]. “MethylCRF,” a novel algorithm based on Conditional Random Fields, integrates additional MRE-seq data on genomic unmethylated regions to predict DNA absolute methylation levels at single-CpG resolution [12]. “QSEA” is a recently developed method that improves on BayMeth by providing a built-in sigmoidal CpG density bias curve without the need for additional experimental data [13]. QSEA(TCGA) curates information on 172 samples from the TCGA lung cancer study [14, 15]. Genomic regions from these samples with mean methylation > 90% serve as a fully methylated control sample. QSEA(blind) estimates CpG density bias based on empirical knowledge. Both versions of QSEA fit a sigmoidal CpG density bias curve, which is incorporated in a Bayesian model to derive the absolute CpG methylation level.

Despite the extensive development of computational methods for inferring absolute CpG methylation levels from DNA enrichment data, there is still room for improving accuracy.

## Methods

### Two aspects for improvement

**Fig. 1:**
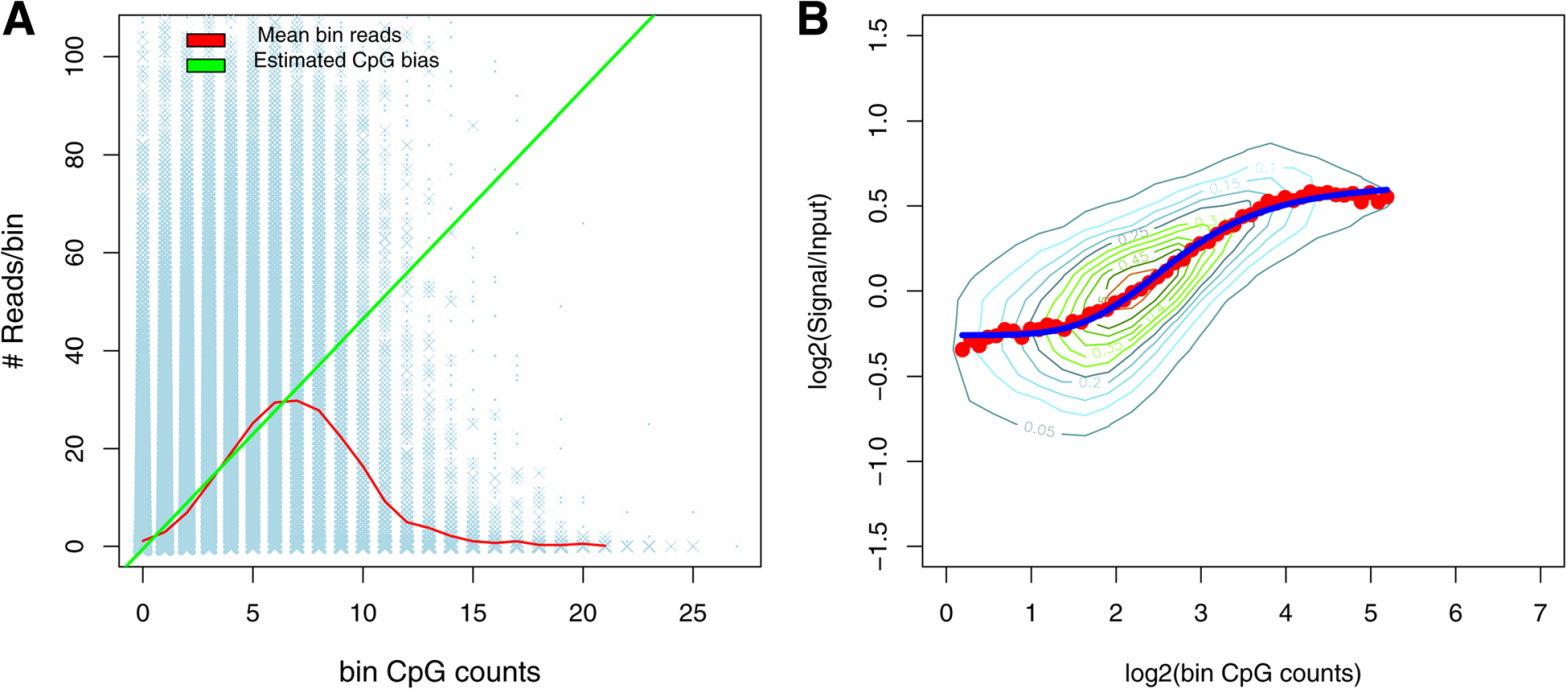
MEDME experiment versus MEDIPS calibration plot. **a** Calibration plot from the MEDIPS method to estimate CpG density bias. The blue stripes indicate grouped bin reads vs. corresponding bin CpG counts. The relationship between the means of bin reads and bin CpG counts are shown by the red bell-curve. The green line represents fitting a simple linear regression model of the relationship from low CpG density regions. **b** The sigmoidal relationship between the MeDIP-ChIP enrichment signal and CpG density was revealed by MEDME in log-scale. The red dots signify the median enrichment signal within a 1 k bp window across the dynamic range of numbers of methylated CpGs

MEDIPS estimates the CpG density effect from MeDIP-seq data itself, without requiring an experimental control sample. Because CpGs are mostly methylated at low CpG density regions and are hypo- or un-methylated at high CpG density regions, MEDIPS uses low CpG density regions as its fully methylated control. For high CpG density regions, the MeDIP enrichment signal decreases significantly due to a decreasing methylation level that overrides the CpG density effect. To estimate the CpG density effect for all regions, MEDIPS fits a linear regression model for the means of the MeDIP enrichment signal at low CpG density regions and extrapolates the fitted line to high CpG density regions (Fig. 1a, green line). The fitted line is the estimated CpG density bias curve.

However, application of MEDME reveals that the CpG density effect is not linear but sigmoidal (Fig. 1b). The assumption of a linear CpG density effect used in “MEDIPS” does not take into consideration the saturation effect of methyl-CpG binding at high CpG density regions, which leads to overestimation and overcorrection of the CpG density bias at these regions. Therefore, the incorporation of a nonlinear model for CpG density estimation in MEDIPS is likely to improve the inference.

**Fig. 2:**
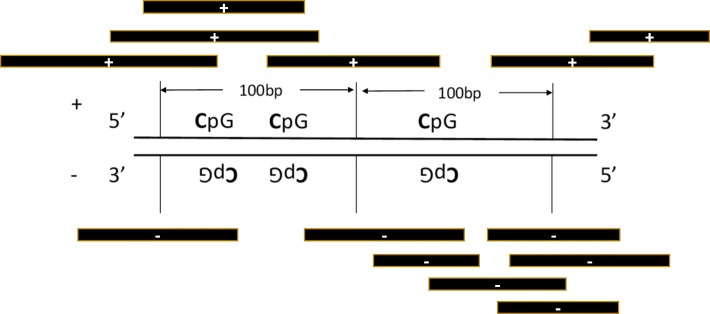
Illustration of counting bin reads. A sliding bin of 100 bp divides the genome and the number of reads that fall in the bins are assigned as bin reads. Bin reads measure fragmented DNA enrichment for loci. Mapped reads include DNA strand information and are usually combined for bin counts for loci. In our method, bin reads are counted for the positive and negative DNA strands separately

**Fig. 3:**
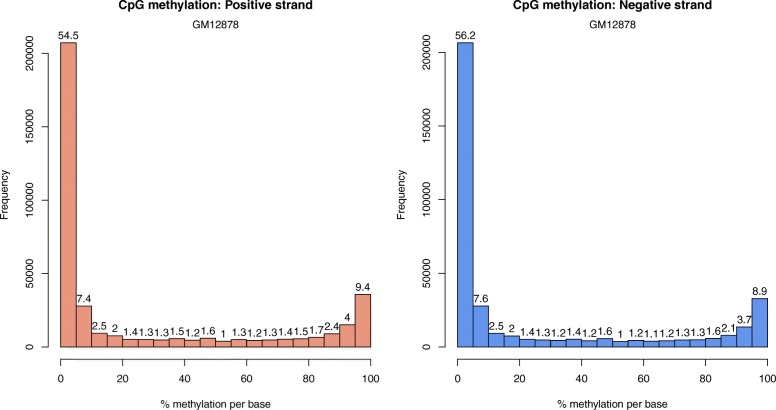
Histograms illustrating the distribution of methylation levels of cytosine within CpG dinucleotides on the positive and negative DNA strands (using RRBS data from cell line GM12878)

In addition, none of the current methods consider the effects of asymmetric CpG methylation, i.e., methylated cytosine within “CG” dinucleotides on one DNA strand and un-methylated adjacent cytosines within the “GC” context (or still “CG” from 5′ to 3′) on the other DNA strand (Fig. 2). Our investigation of RRBS data for the cell line GM12878 showed that cytosine methylation within CpG on the positive and negative DNA strands is different (Fig. 3). The bin-wise discordance of methylation levels between the two strands (by taking the mean of all cytosines within the bin) increases with increasing bin size. Six chromosomes (1, 2, 11, 12, 21, 22) were selected to represent chromosomes of large, medium, and small size (Table 1). The complete comparison is provided in the Additional file 1: Table S1.

**Table 1:** Pearson correlation coefficients of bin methylation level for positive and negative DNA strands at various bin sizes (bp). Cell line: GM12878, Data: RRBS

bin size	chr1	chr2	chr11	chr12	chr21	chr22
25	0.96	0.95	0.96	0.96	0.96	0.96
50	0.93	0.92	0.93	0.94	0.94	0.94
100	0.88	0.87	0.87	0.89	0.89	0.88
150	0.86	0.86	0.85	0.88	0.85	0.85
200	0.86	0.85	0.84	0.87	0.85	0.84

Based on the above analysis, we developed our method, MeDEStrand (inferring genome-wide absolute methylation level from DNA enrichment data utilizing strand-specific processing), for the inference of absolute methylation levels. MeDEStrand improves on the MEDIPS approach in two ways:

1. Uses a logistic regression model for the estimation of CpG density effect. The upper asymptote of the sigmoid function is more suitable for modeling the saturation point of methyl-CpG-binding for high CpG density regions.
2. Estimates and corrects CpG density bias from enrichment bin reads for the positive and negative DNA strands separately to take into consideration the effect of asymmetric CpG methylation of each strand.

### Experimental data for evaluation

Hg19 mapped MeDIP-seq and RRBS data were downloaded from the ENCODE Consortium [16] and GEO [17]. RRBS data were used as a “gold standard” for method validation and comparison to previously published methods.

Though several cell types have MeDIP-seq and corresponding RRBS data available, we chose to use data from immortalized cell lines and primary cells to limit variation in CpG methylation due to the heterogeneity of tissue. We selected two immortalized cell lines (GM12878 and K562) and two types of primary cells (foreskin fibroblasts and mammary epithelial cells) for our study to ensure high consistency in MeDIP-seq and RRBS data. Since DNA methylation is a highly dynamic and transient epigenetic event [18–20], cells with closely matching datasets were selected to ensure high confidence of our MeDEStrand results.

Unmapped raw RRBS data (i.e., the .sra format file) enabled us to retrieve the methylation value for every cytosine within CpG dinucleotides so that the strand-specific methylation information could be investigated. The SRA Toolkit [21], samtools [22], Bismark [23], Bioconductor packages methylKit [24] and IRanges [25], R package stats [26] were used in the data analysis.

### Model and the algorithm

**Fig. 4:**
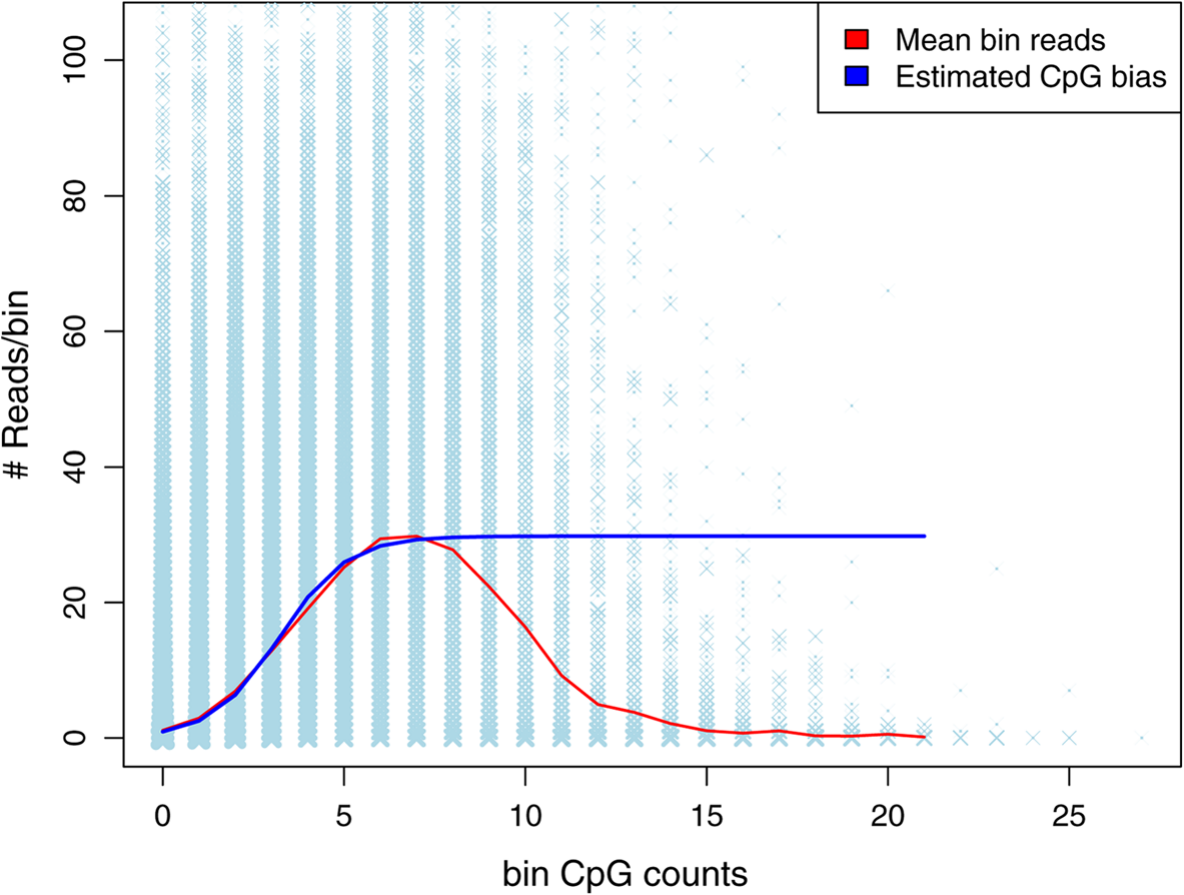
The fitted sigmoid function by MeDEStrand. A logistic regression model is fitted to estimate CpG bias using information from low CpG density regions in MeDEStrand (blue line). The blue stripes indicate grouped bin reads vs. corresponding bin CpG counts. The relationship between the means of bin reads and bin CpG counts are shown by the red line

We utilized the low CpG density regions of the MeDIP-seq data as the fully methylated control. We used a logistic regression model to describe the means of the enrichment signal (in terms of bin reads) as a function of the corresponding number of CpGs. We let the upper asymptote be the maximum mean observed, thus the bin reads corresponding to the high CpG regions are not included in the model fitting. The fitted curve that extended to high CpG density regions was the estimated CpG bias curve for all regions (Fig. 4, blue line).

We modeled the bin reads (*y*) to be the CpG methylation-induced enrichment signal (*M*_*CpG*_) multiplied by the CpG density effect (*f*(*n*_*CpG*_)). The latter is a function of the number of CpGs within the bin:

1 $$ y={M}_{CpG}.f\left({n}_{CpG}\right) $$

*f*(*n*_*CpG*_) was estimated from MeDIP-seq data (see details under **the algorithm workflow** section, below). By dividing *f*(*n*_*CpG*_) from both sides of (1), we obtained the corrected enrichment signal that is related to the methylation level:

2 $$ {y}^{\prime }={M}_{CpG} $$

Heuristically, log-transformation before scaling further improves the accuracy. We thus log-transformed *y*^′^ to produce *y*^"^:

3 $$ {y}^{"}=\log \left({M}_{CpG}\right) $$

We normalized *y*^"^ by $\frac{y^{"}-{y}_{min}^{"}}{y_{max}^{"}-{y}_{min}^{"}}$ to generate values between 0 to 1 as the absolute methylation levels for the bins. ${y}_{min}^{"}$ and ${y}_{max}^{"}$ correspond to the minimum and maximum values of *y*^"^, respectively. The above steps were performed for the positive and negative DNA strands separately to take into consideration the effect of asymmetric CpG methylation of each strand. The mean of the inferred absolute methylation levels from both strands is reported as inferred absolute methylation level for the loci.

### The algorithm workflow

The complete steps of our method are as follows:

**Input**: MeDIP-seq data.

**Output**: bin-based absolute methylation levels.

**For** each DNA strand, **do**

Divide the given chromosome(s) into user-specified bin size (recommend 50 or 100 bp). Count bin reads for the positive and the negative DNA strand separately.

1. Group bins with the same CpG counts and sort in the ascending order.
2. Let bin CpG count *n*_*CpG*_ of groups be the explanatory variable and the mean bin reads $\overline{y}$of the groups be the response variable. Fit the logistic regression model.

log($$ \frac{\overline{y}/{y}_{max}}{1-\overline{y}/{y}_{max}} $$) = *β*_0_ + *β*_1_ ∙ *n*_*CpG*._

*y*_*max*_: the maximum of $\overline{y}$

3) Divide bin reads by corresponding estimated CpG density effect

$$ f\left({n}_{CpG}\right)=\frac{\exp \left({\beta}_0+{\beta}_1\bullet {n}_{CpG}\right)}{1+\exp \left({\beta}_0+{\beta}_1\bullet {n}_{CpG}\right)} $$

from the fitted model in 2).

4) Log transform corrected bin reads from 3).
5) Scale bin reads from 4) to values between 0 to 1, and report them as the inferred strand-specific bin-based absolute methylation level.

**End**

Merge inferred bin absolute methylation values from the positive and negative DNA strands by taking the mean. Report them as genome-wide bin-based absolute methylation levels.

The function “glm()” from the R package “stats” was used to fit the logistic regression model.

## Results

### Criteria for evaluation

To evaluate the accuracy of inferred methylation levels, we used RRBS data as the gold standard, calculating the mean of CpG methylation levels provided by RRBS within each bin as the true methylation level for the bin. From the ENCODE protocol, each CpG from the RRBS data was covered by at least 10 reads. We kept all RRBS CpGs without further filtering, since a 10-read coverage would give good confidence. For the validation, we kept bins that had at least 4 RRBS CpGs, as this would provide methylation information from at least two non-adjacent cytosines (Fig. 2).

We used the Pearson correlation coefficient (PCC) and Spearman correlation coefficient (SCC) as the criteria to measure the agreement between the inferred methylation levels from the MeDIP-seq data and the true methylation levels from the RRBS data. PCC and/or SCC were used as the primary criteria for method evaluation and comparison to previous studies [2, 7, 9, 11, 12]. While PCC assesses linear relationships between two sets of data, SCC uses the ranks of the values and assesses monotonic relationships regardless linear or not. Higher PCC and/or SCC indicated higher concordance. We included both PCC and SCC in order to more fairly compare the methods and make more reliable conclusions.

### Comparison with other methods

To assess the performance of our method MeDEStrand, we compared it with three other state-of-the-art methods MEDIPS, BayMeth, and QSEA. For each method, we chose the version(s) that could be run using the available data (i.e., MeDIP-seq data) to infer absolute methylation levels. For BayMeth, we used the version “SssI-free”, since a fully methylated experiment control sample preferred by BayMeth was not available. Of the three versions of QSEA, we chose two, QSEA(TCGA) and QSEA(blind), which do not require additional experimental data. The third version QSEA(BS) requires a fully methylated experimental control sample, which was not available. For MEDIPS and our method MeDEStrand, no additional experimental data is required. The method methylCRF was not included, as it was designed for paired-end sequencing reads while the data from ENCODE are from single-end sequencing.

We ran these methods on the 22 chromosomes (from chromosome 1 to 22) of the MeDIP-seq data to infer absolute methylation levels. Among the four cell types included in our study, GM12878, K562, and mammary epithelial cells were from female donors and cell type foreskin fibroblasts were from male donors. To account for gender differences, we did not include the Y chromosome data in our analysis. We also did not include the X chromosome, since we found that the GM12878 MeDIP-seq data for the X chromosome was corrupted.

Bin size is an important parameter that limits the resolution of inferred absolute methylation levels. In previous studies, bin sizes of 50 bp or 100 bp were deemed high resolution. Since all the methods in our comparison infer bin-based absolute methylation levels, we chose bin sizes of 25 bp, 50 bp and 100 bp to examine the accuracy of each method.

**Fig. 5:**
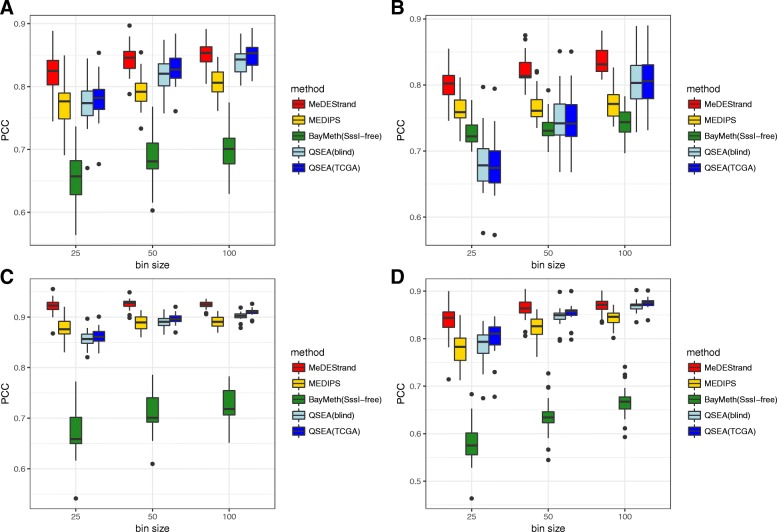
Comparison of the CpG methylation inference methods. Pearson correlation coefficient (PCC) between MeDIP-seq and RRBS data calculated for four cell types: **a** GM12878; **b** K562; **c** foreskin fibroblasts; and **d** mammary epithelial cells. Y-axis shows the PCC values. X-axis shows the varying parameter bin size from 25 bp to 100 bp. Boxplot illustrates the variation of PCC across the 22 chromosomes

Figure 5 compares the performance of each method based on PCC. BayMeth did not perform well at any bin size for any of the cell types. This may be due to the lack of a fully methylated experimental control sample needed by the BayMeth model in order to make a good inference. Comparing the results for all cell types and bin sizes, we concluded that MeDEStrand has the best performance with regards to the median value of PCCs across the 22 chromosomes. We also noticed that QSEA(blind) and QSEA(TCGA) had a similar performance as MeDEStrand at bin size 100 bp. However, the QSEA PCCs had greater variation across the 22 chromosomes in most of the cases. For mammary epithelial cells, QSEA(TCGA) performed slightly better than MeDEStrand.

**Fig. 6:**
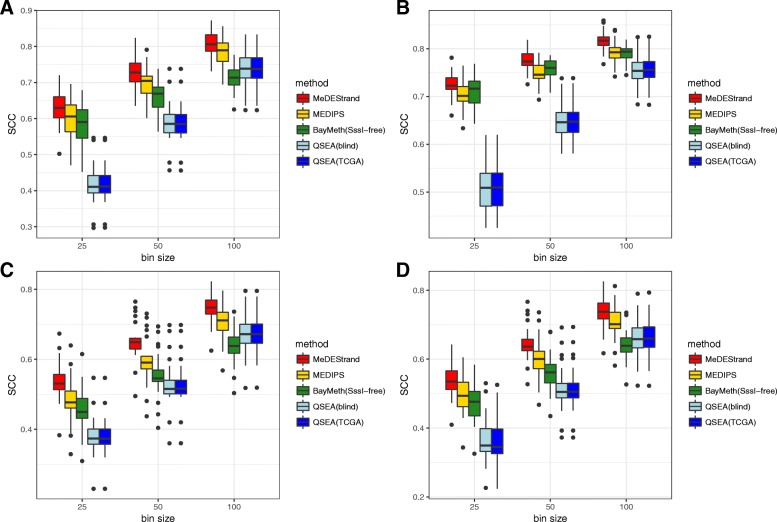
Comparison of the CpG methylation inference methods. Spearman correlation coefficient (SCC) between MeDIP-seq and RRBS data calculated for four cell types: **a** GM12878; **b** K562; **c** foreskin fibroblasts; and **d** mammary epithelial cells. Y-axis shows the SCC values. X-axis shows the varying parameter bin size from 25 bp to 100 bp. Boxplot illustrates the variation of SCC across the 22 chromosomes

Figure 6 summarizes the performance of each approach based on SCC. MeDEStrand again showed the best performance among all methods at all bin sizes for all cell types. QSEA(blind) and QSEA(TCGA) had reduced performance at bin sizes of 25 bp and 50 bp. Noticeably, based on SCC, all methods have lower values.

Finally, the processing time for one sample (including the time to import data) ranged from approximately 25 min to 3 h, when run on a MacBook Pro laptop with 2.5GHz quad-core Intel Core i7 and 16G RAM. MeDEStrand had one of the shortest processing times among all the methods (~ 25 min). We deemed that the processing time is not a key criterion for method comparison since all methods provided reasonably fast processing.

Taken together, we demonstrated that, compared to several other methods, MeDEStrand is a robust method to infer genome-wide absolute methylation levels at bin sizes of 25 bp, 50 bp, and 100 bp. Smaller bin sizes provided higher resolution. MeDEStrand has been implemented as a R package and is freely available for download from GitHub: https://github.com/jxu1234/MeDEStrand.git.

### Improving accuracy using strand-specific reads processing and a sigmoid function to estimate CpG bias

As described previously, MeDEStrand uses a sigmoid function to estimate CpG bias from the methylation enrichment signal. In addition, MeDEStrand estimates and corrects CpG bias for the positive and negative DNA strands separately, and then reports the average of the inferred strand-specific absolute methylation levels as the absolute methylation levels for the loci. We investigated the unique contribution of these two aspects of MeDEStrand to its observed performance in the comparative study.

We constructed a modified version of MEDIPS, namely, “MEDIPS(strand-processing)” which uses the same algorithm as MEDIPS except that process reads mapped to the positive and negative DNA strands separately. To illustrate the impact of this step on inferring CpG methylation in regions of different CpG density, we divided all bins into four categories based on their CpG counts. The first category consisted of bins with CpG counts from the minimum to the 1st quartile, corresponding to “low” CpG density regions. The second category consisted of bins with CpG counts from the 1st quartile to the median, corresponding to “lower-medium” CpG density regions. The third category consisted of bins with CpG counts from the median to the 3rd quartile, corresponding to “higher-medium” CpG density regions. The last category consisted of bins with CpG counts from the 3rd quartile to the maximum, corresponding to “high” CpG density regions. These four categories thus represented different DNA CpG density compositions within the bins. We report here the results using cell line GM12878 at bin size 100 bp as an example.

**Fig. 7:**
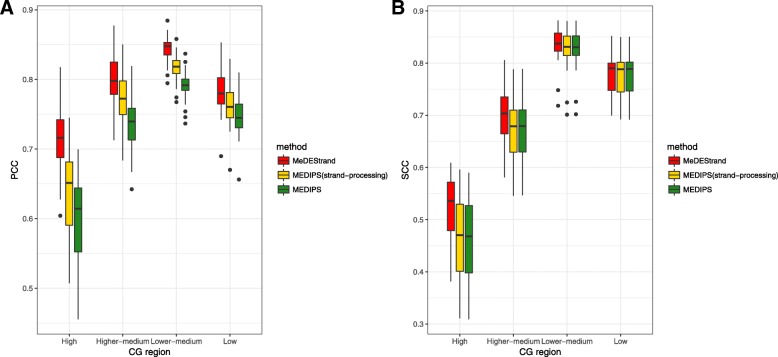
Comparison of the methods MeDEStrand and MEDIPS as well as its modified version MEDIPS(strand-processing). **a** Pearson correlation coefficient (PCC) is calculated for different CpG density regions. **b** Spearman correlation coefficient (SCC) is calculated for different CpG density regions. The MeDIP-seq and RRBS data of GM12878 cells were used for the demonstration

Figure 7 compares the performance of MEDIPS, MEDIPS(strand-processing), and MeDEStrand at different CpG density regions evaluated by the Pearson correlation coefficient (PCC) and the Spearman correlation coefficient (SCC). Noticeably, MEDIPS(strand-processing) had improved performance compared to MEDIPS at all CpG density regions based on the PCC but not the SCC criterion. The result demonstrates that by merely adding the procedure for strand-specific processing, we were able to improve the overall performance of MEDIPS at least by the PCC criterion. Meanwhile, MeDEStrand was more robust and improved the accuracy under both criteria. We note that for all the previous methods, bin reads were counted by combining reads mapped to the same loci, which discounts any strand-specific information. However, we showed that strand-specific processing improved the accuracy of inference.

Our analysis also revealed that the highest correlation values (both the PCC and the SCC) occur in lower-medium CpG density regions and that the values decrease when the regional CpG density either decreases or increases. This may be because at low CpG density regions, the inference of absolute methylation levels is often more difficult when using enrichment-based methods than when using bisulfite conversion methods. At lower sequencing depth, the lack of methylation cannot be distinguished from the lack of coverage, due to the stochastic nature of read coverage from the enrichment-based methods. At the high CpG density regions, the performance of MEDIPS and MEDIPS(strand-processing) were significantly deteriorated. We also saw the largest variation in the correlation values across the 22 chromosomes for these two methods. By comparison, MeDEStrand had higher values, with much less variation. This category corresponds to the high CpG density regions where MeDEStrand showed the most improvement compared to MEDIPS and MEDIPS(strand-processing). These findings demonstrate the advantage of a logistic regression model over a linear model to estimate CpG bias, with its upper asymptote taking into account the saturation effect of methyl-CpG binding.

The MeDEStrand approach had a synergistic effect on improvement of inferring CpG methylation levels, utilizing strand-specific processing in addition to CpG bias estimation and correction by a sigmoid function to achieve better performance than MEDIPS(strand-processing). It should be noted, however, that at the high CpG density regions, less accurate inference is made by all of the methods tested.

### A further look into strand-specific processing

We utilized MEDIPS and MEDIPS(strand-processing) to further inspect how strand-specific processing improves the overall performance. The latter differs from the former only by the additional step to process reads mapped to the positive and negative DNA strands separately.

As with previous methods, MEDIPS counts bin reads by combining reads mapped to the same genomic loci, and strand information is lost. For MEDIPS(strand-processing), reads mapped to the positive and negative DNA strands are counted separately, i.e., strand-specific bin reads. The same loci bins may or may not have the same reads for the positive and negative DNA strands (Fig. 2). We observed that approximately 40% of genomic coverage contains different bin reads for the positive and negative DNA strands. We wondered if the asymmetric bin reads or merely the procedure of strand-specific processing (i.e., irrelevant to the asymmetry of the bin reads) contributes to the improvement in inferring CpG methylation levels.

To investigate this question, we devised a counting scheme that eliminates the asymmetric bin reads for the positive and negative DNA strands. That is, we divided each bin read of MEDIPS evenly and re-assigned the halved bin reads for bins residing on the positive and negative DNA strands. Note that this re-assignment had no effect on the outcome of MEDIPS, since combined reads for the loci remain the same. However, for MEDIPS(strand-processing), any asymmetry of the bin reads was eliminated. We re-ran MEDIPS(strand-processing) and observed no improvement compared to MEDIPS. In fact, MEDIPS and MEDIPS(strand-processing) had the same performance. This result suggests that MEDIPS can be viewed as a special case of MEDIPS(strand-processing), whereby bin reads for the positive and negative DNA strands are equal.

We also investigated the correlations (both the PCC and the SCC) for each DNA strand to see if the improvement of strand-specific analysis is attributed to the strand difference of DNA methylation. To do so, we used the same bins of size 100 bp (see **Results** section, subsection **Criteria for evaluation**) without further filtering to keep the bin numbers the same for each DNA strand and between the methods, and we then calculated the correlations between the inferred strand-specific bin methylation level by MeDEStrand with the RRBS CpGs that fell in the bins from the same DNA strand. Note that the inferred strand-specific bin methylation level is an intermediate result from MeDEStrand (see **Methods** section, subsection **The algorithm workflow**). We compared the correlations with those of MEDIPS and MeDEStrand. Interestingly, we see the gradual increment of correlations in the order of MEDIPS, MeDEStrand (strand-specific), and MeDEStrand, although the improvement is not always statistically significant. The result from the selected chromosomes of cell line GM12878 (as representative) by the SCC criterion is shown in Table 2. The complete result on 22 chromosomes of the four cell types by both the PCC and the SCC criteria is provided in the Additional file 2: Table S2 and Additional file 3: Table S3.

**Table 2:** Spearman correlation coefficients (SCC) between the inferred bin methylation level from different methods and the RRBS data. Cell line: GM12878, bin size: 100 bp

	chr1	chr2	chr11	chr12	chr21	chr22
MEDIPS	0.7419	0.7599	0.7621	0.7566	0.8556	0.8127
MeDEStrand (only positive strand)	0.7837	0.7814	0.7933	0.7913	0.8679	0.824
MeDEStrand (only negative strand)	0.7827	0.7836	0.7939	0.7959	0.8667	0.8271
MeDEStrand	0.7858	0.7853	0.7943	0.7945	0.8719	0.8277

Thus, we demonstrated improvement in CpG methylation inference due to strand-specific processing, which takes into account asymmetric bin reads for the positive and negative DNA strands.

### Some additional investigation

In the procedure used in MeDEStrand to estimate CpG density bias, the means of bin reads show a normal curve (Fig. 4, red curve). However, MeDEStrand does not correct CpG density bias by the normal curve. In Additional file 4, we explained why and proved our point by conducting a computational experiment. The result is shown in Additional file 5: Figure S1 and Additional file 6: Figure S2. Additionally, we re-evaluated the performance of the methods using a WGBS data of cell line GM12878. The result is shown in Additional file 7: Figure S3.

## Discussion

We developed and demonstrated the improved performance of MeDEStrand in inferring CpG methylation levels based on MeDIP-seq enrichment data, compared to various other computational approaches. MeDEStrand can be applied to other enrichment-based sequencing data such as MethylCap-seq/MDB-seq, where the main bias also comes from CpG density.

The MeDIP-seq data from ENCODE was prepared from non-strand-specific libraries; however, in the IP step, an anti-methylcytosine antibody is used to pull down methylated single-stranded DNA fragments. By contrast, MethylCap/MBD-seq utilizes the MBD2 protein’s methyl-CpG binding domain to capture methylated double-stranded DNA fragment [1, 5, 27]. In this sense, the IP step is “strand-specific” for MeDIP-seq and “non-strand-specific” for MethylCap/MBD-seq. We are unclear if the improvement in CpG methylation inference from strand-specific processing is related to this fact or merely a result of more accurate estimation of the CpG density effect when data is processed in a strand-specific way. The answer to this question will require further study of datasets generated using “strand-specific” libraries.

Although MeDEStrand showed better performance than MEDIPS at all CpG density regions, MeDEStrand was less accurate at high CpG density regions (Fig. 7). Future work will need to identify the cause and improve accuracy for these regions.

As described previously, MEDME, QSEA, and MeDEStrand all utilize sigmoid functions to describe the CpG density effect. Although based on different platforms (microarray vs. high-throughput sequencing), their main differences lie in how a fully methylated control sample is constructed for the estimation of the CpG density effect. MEDME generates a fully methylated control sample experimentally, whereas QSEA constructs a virtual fully methylated control sample based on curated information from 172 samples from the TCGA lung cancer study. MEDME and QSEA do not estimate a CpG density bias curve for each sample; rather, the estimated CpG density bias curve is built into the package and used generically for all samples. Since our MeDEStrand method estimates the CpG density effect from MeDIP-seq data itself, the estimation is sample-specific.

Previous methods showed a performance gain as a consequence of explicitly modeling copy number variation (CNV), which directly affects read density [28, 29]. In a 2013 paper, 37 tools were reviewed to identify whole-genome CNVs based on various computational strategies [30]. Further improvement may be possible by incorporating a suitable CNV modeling strategy into our MeDEStrand approach.

DNA methylation occurs mainly at the C5 position of cytosine within CpG dinucleotides in somatic cells and non-CpG cytosine in plants and embryonic stem cells in mammals [31, 32]. For the somatic cell lines, DNA methylation occurs predominantly at CpG sites. By contrast, 25% of DNA methylation in embryonic stem cells occurs at CHG and CHH sites [33]. Unlike enrichment methods based on the MBD protein, which only binds to the double-stranded DNA methylated at CpG sites, the antibody-based MeDIP-seq method also captures CHG and CHH methylation sites. Current methods that infer DNA absolute methylation only consider CpG methylation effects for the enrichment [8–13]. To our best knowledge, no method has incorporated CHG and CHH methylation effects. For embryonic stem cells or those cells where a significant amount of DNA methylation occurs at non-CpG sites, CHG and CHH methylation should be taken into consideration for further improvement in the inference of DNA absolute methylation levels for MeDIP-seq data.

## Conclusions

MeDEStrand outperformed the existing state-of-the-art methods for CpG methylation inference from DNA enrichment data at high resolutions (25 bp, 50 bp, and 100 bp bin sizes) based on evaluation of four independent datasets. In addition, MeDEStrand achieved high accuracy when only using MeDIP-seq data. Thus, MeDEStrand does not require additional experimental data to achieve good performance, unlike BayMeth method. We conclude that MeDEStrand may be a particularly useful tool to analyze data from the public repository where additional experimental data are not always available. The observed improvement in CpG methylation inference with MeDEStrand compared to other methods was achieved by processing asymmetric bin reads in a strand-specific manner. Future studies will explore asymmetric bin reads as an area of further methodologic development.

## Additional files

Additional file 1: *Correcting CpG density bias by the normal curve* and *Using WGBS data for validation*. (DOCX 16 kb)

Additional file 2: **Table S1.** Pearson correlation coefficient of bin methylation level for the positive and negative DNA strand at various bin size (bp). Cell line: GM12878, Data: RRBS. (XLSM 32 kb)

Additional file 3: **Table S2.** Pearson correlation coefficient between the inferred bin methylation level and the RRBS data, bin size: 100 bp. (XLSM 38 kb)

Additional file 4: **Table S3.** Spearman correlation coefficient between the inferred bin methylation level and the RRBS data, bin size: 100 bp. (XLSM 38 kb)

Additional file5: **Figure S1.** Comparison of the methods MEDIPS_normal and MEDIPS. Pearson correlation coefficient (PCC) between MeDIP-seq and RRBS data calculated for four cell types: **A** GM12878; **B** K562; **C** foreskin fibroblasts; and **D** mammary epithelial. Y-axis shows the PCC. X-axis shows the varying parameter bin size from 25 bp to 100 bp. Boxplot illustrates the variation of PCC across the 22 chromosomes. (PDF 8 kb)

Additional file 6: **Figure S2.** Comparison of the methods MEDIPS_normal and MEDIPS. Spearman correlation coefficient (SCC) between MeDIP-seq and RRBS data calculated for four cell types. **A** GM12878; **B** K562; **C** foreskin fibroblasts; and **D** mammary epithelial cells. Y-axis shows the SCC values. X-axis shows the varying parameter bin size from 25 bp to 100 bp. Boxplot illustrates the variation of SCC across the 22 chromosomes. (PDF 10 kb)

Additional file 7: **Figure S3.** Comparison of all methods for inferring CpG methylation levels based on DNA enrichment data. **A** Pearson (PCC) and **B** Spearman correlation coefficients (SCC) between the MeDIP-seq and WGBS data for the GM12878 cell line are calculated. Y-axis shows the PCC or SCC values. X-axis shows the varying parameter bin size from 25 bp to 100 bp. Boxplot illustrates the variation of PCC and SCC across the 22 chromosomes. (PDF 9 kb)

## Abbreviations

bp: base pair
kb: A kilobase pair, i.e., 1000 base pairs
PCC: Pearson correlation coefficient
RRBS: reduced-representation bisulfite sequencing
SCC: Spearman correlation coefficient
WGBS: whole-genome bisulfite sequencing
