# Supplementary material for: MeDEStrand: an improved method to infer genome-wide absolute methylation levels from DNA enrichment data
Source: BMC Bioinformatics. 2018 Dec 22;19:540. doi: 10.1186/s12859-018-2574-7 (PMC6303941; doi:10.1186/s12859-018-2574-7)
Supplement: Supplementary file 1 — Correcting CpG density bias by the normal curve and Using WGBS data for validation. (DOCX 16 kb) [file 12859_2018_2574_MOESM1_ESM.docx]

**Additional file 1**

***Correcting CpG density bias by the normal curve***

In the procedure used in MeDEStrand to estimate CpG density bias, the means of bin reads show a normal curve (Figure 4, red curve). In immunoprecipitation, DNA fragments with higher CpG density have higher binding affinity. Thus, the decreasing in the normal curve from the midpoint does not reflect a CpG density effect, but rather, is the result of decreasing methylation levels that override the CpG density effect for high CpG density regions, since these regions are mostly hypo- or un-methylated.

To demonstrate, we constructed a modified version of MEDIPS, called MEDIPS_normal, which corrects the CpG density bias by a normal curve. We compared its performance to MEDIPS, and the results are shown in supplementary Figures S1 and S2. PCCs and SCCs between MeDIP-seq and RRBS data are significantly lower for MEDIPS_normal compared to MEDIPS, except for the K562 cell line, which may have a different compositional effect. Thus, the normal curve does not estimate CpG density bias correctly and should not be used.

***Using WGBS data for validation***

Various genome-targeted bisulfite-based approaches have been used as the gold standard in research, including Illumina Human Methylation 450k array, RRBS, clonal bisulfate sequencing, and bisulfite PCR-sequencing, etc. All of these methods provide single-base resolution of cytosine methylation status; however, they do not provide broad genome-wide CpG coverage compared to WGBS. Due to the scarcity of WGBS data, we used data from GM12878 cells to compare the various absolute CpG methylation inference methods at a genome-wide scale. We chose bins for which all the methods (except for BayMeth, which covers 75%~80% of these bins) had inferred methylation levels, and that contain at least two non-adjacent cytosines within CpG and are covered by at least 10 reads. The result is shown in supplementary Figure S3.

Notably, the inferred methylation level from all methods showed reduced concordance with the WGBS data compared to the RRBS data. Since RRBS targets genomic regions with moderate to high CpG density, the reduced concordance suggests that accurate inference of absolute DNA methylation level for regions with low CpG density remains challenging. We note that the method MEDIPS_normal, which corrects CpG density bias by the normal curve, showed relatively improved performance. This is because MEDIPS_normal estimates CpG density bias correctly for low CpG density regions, which leads to the improved overall performance when more low CpG density regions are included. Overall, relative performance of the methods does not change under the WGBS context and the method MeDEStrand continued to be one of the best performers.
