# Supplementary figures and images for: MeDEStrand: an improved method to infer genome-wide absolute methylation levels from DNA enrichment data
Source: BMC Bioinformatics. 2018 Dec 22;19:540. doi: 10.1186/s12859-018-2574-7 (PMC6303941; doi:10.1186/s12859-018-2574-7)

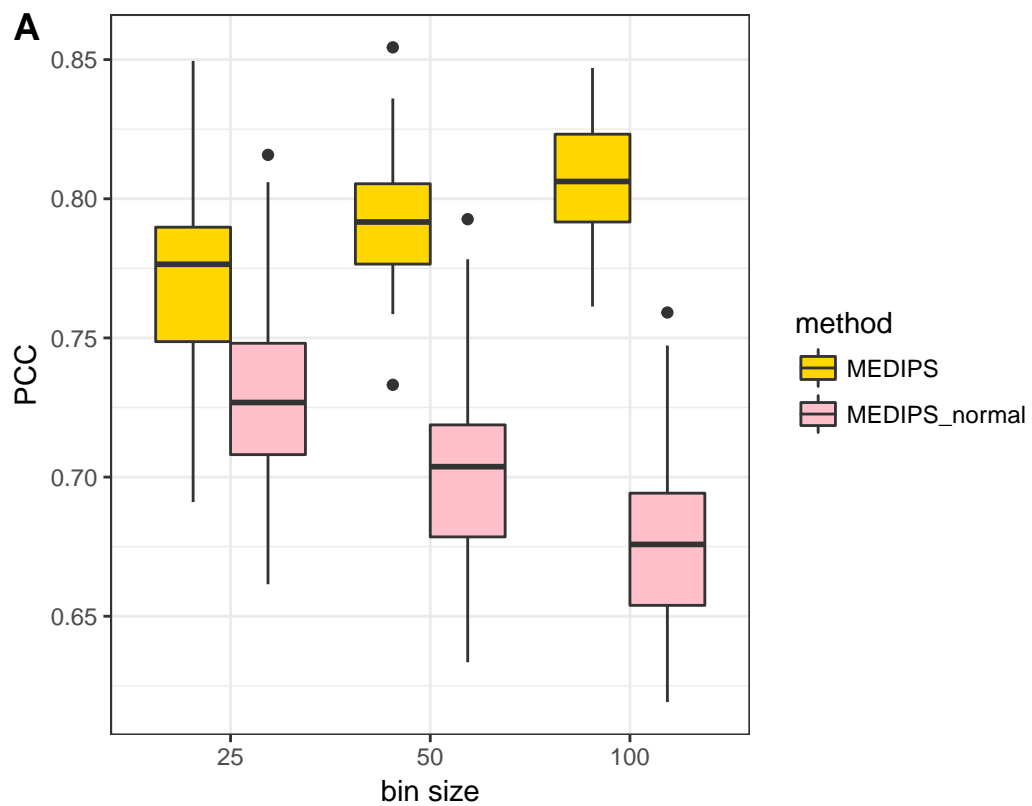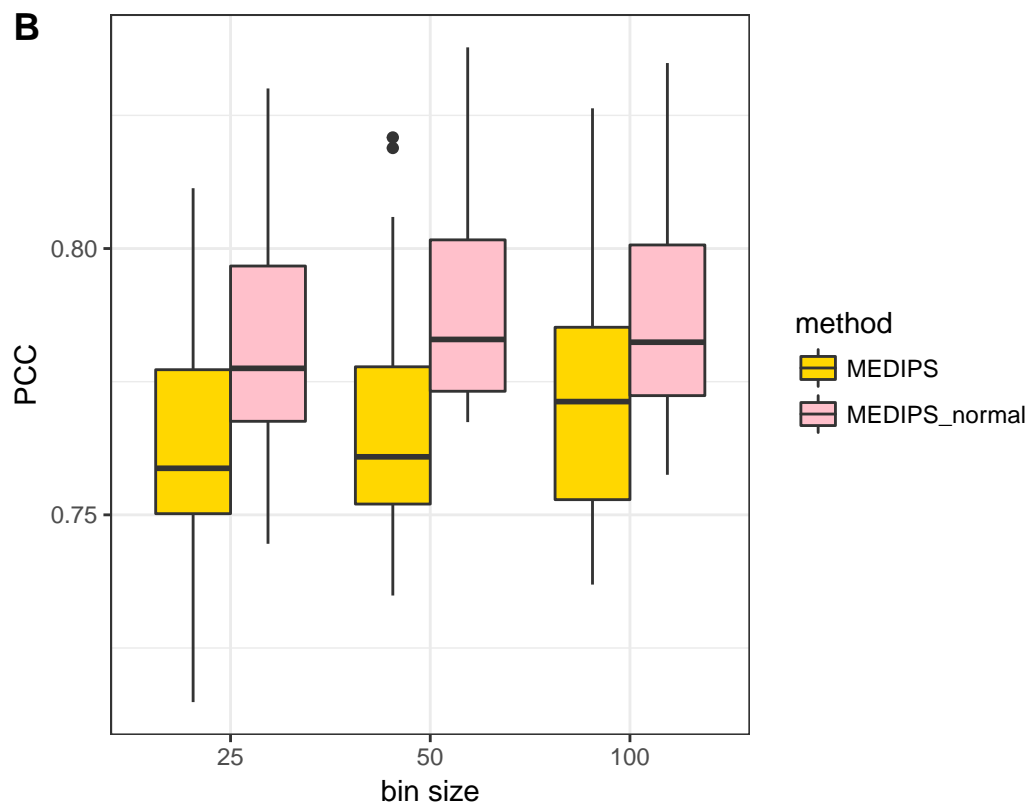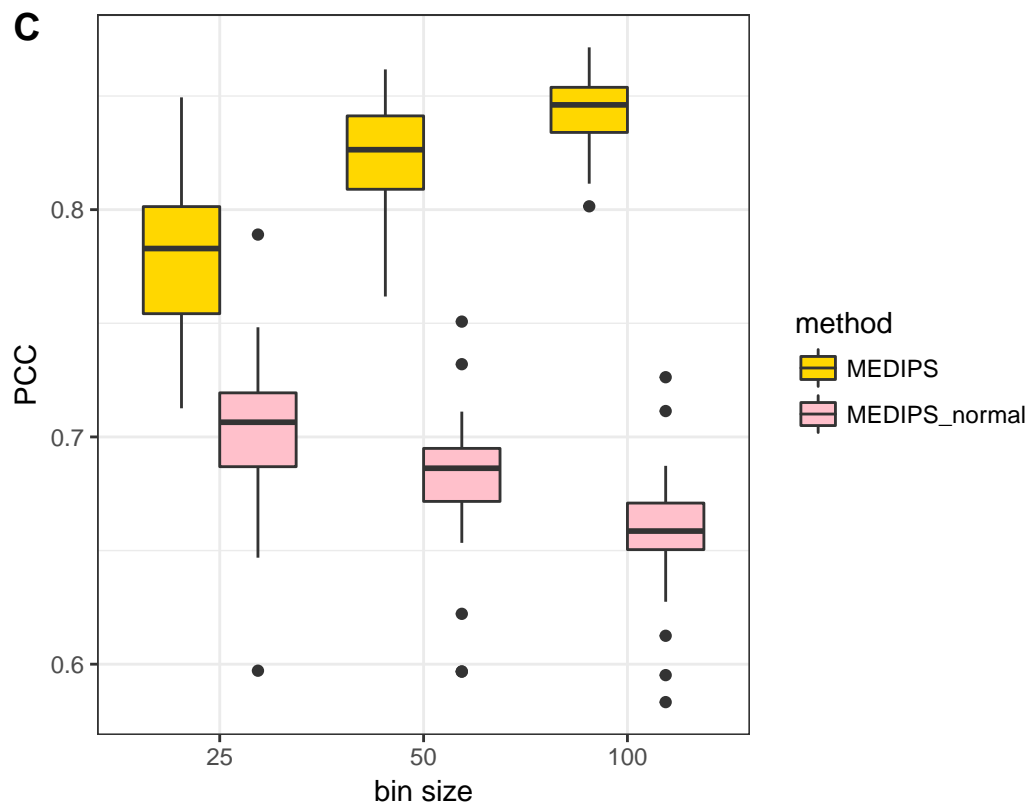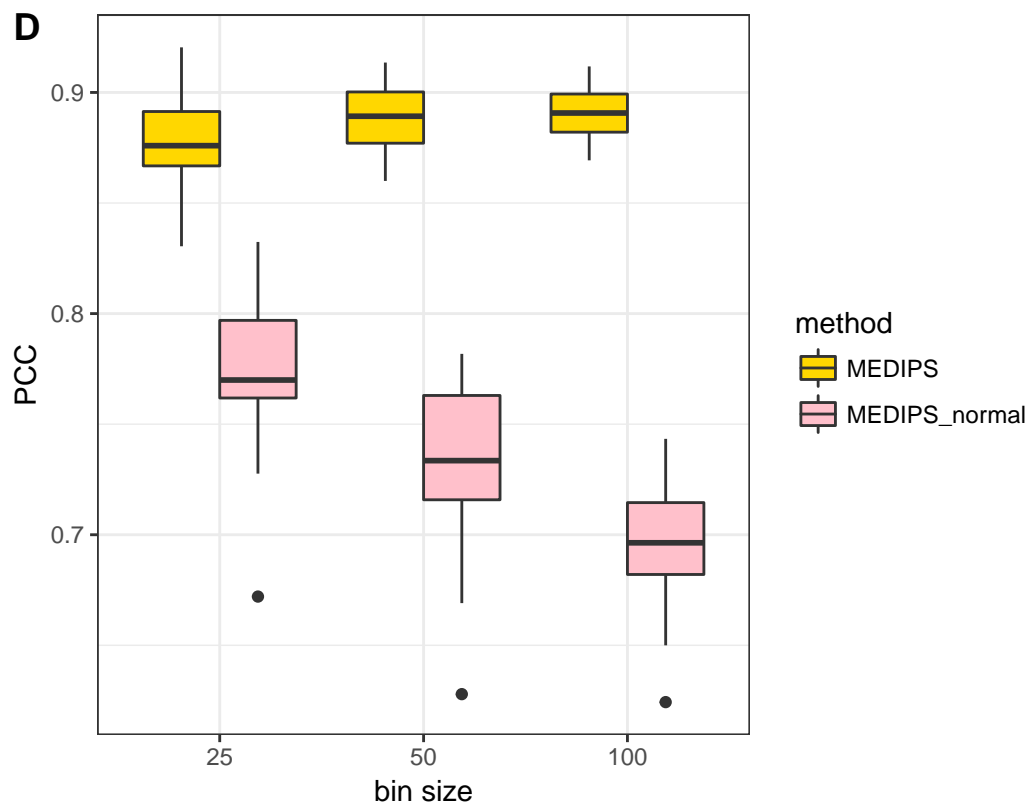

Supplement: Supplementary file 5 — Figure S1. Comparison of the methods MEDIPS_normal and MEDIPS. Pearson correlation coefficient (PCC) between MeDIP-seq and RRBS data calculated for four cell types: A GM12878; B K562; C foreskin fibroblasts; and D mammary epithelial. Y-axis shows the PCC. X-axis shows the varying parameter bin size from 25 bp to 100 bp. Boxplot illustrates the variation of PCC across the 22 chromosomes. (PDF 8 kb) [file 12859_2018_2574_MOESM5_ESM.pdf]

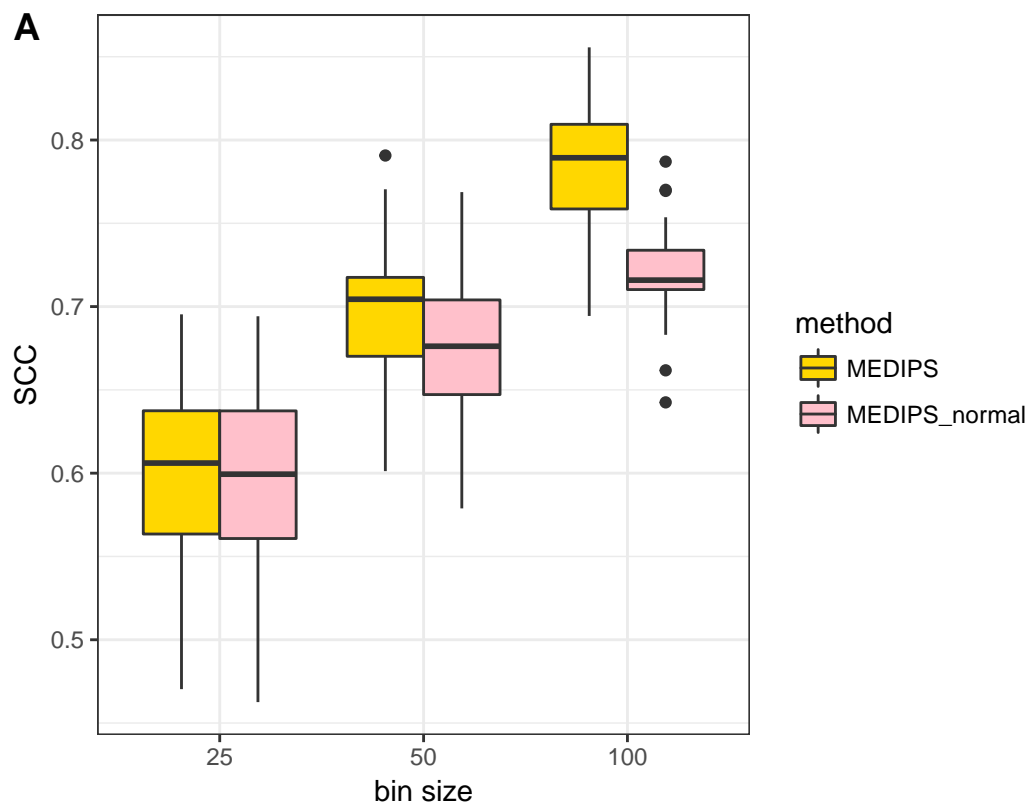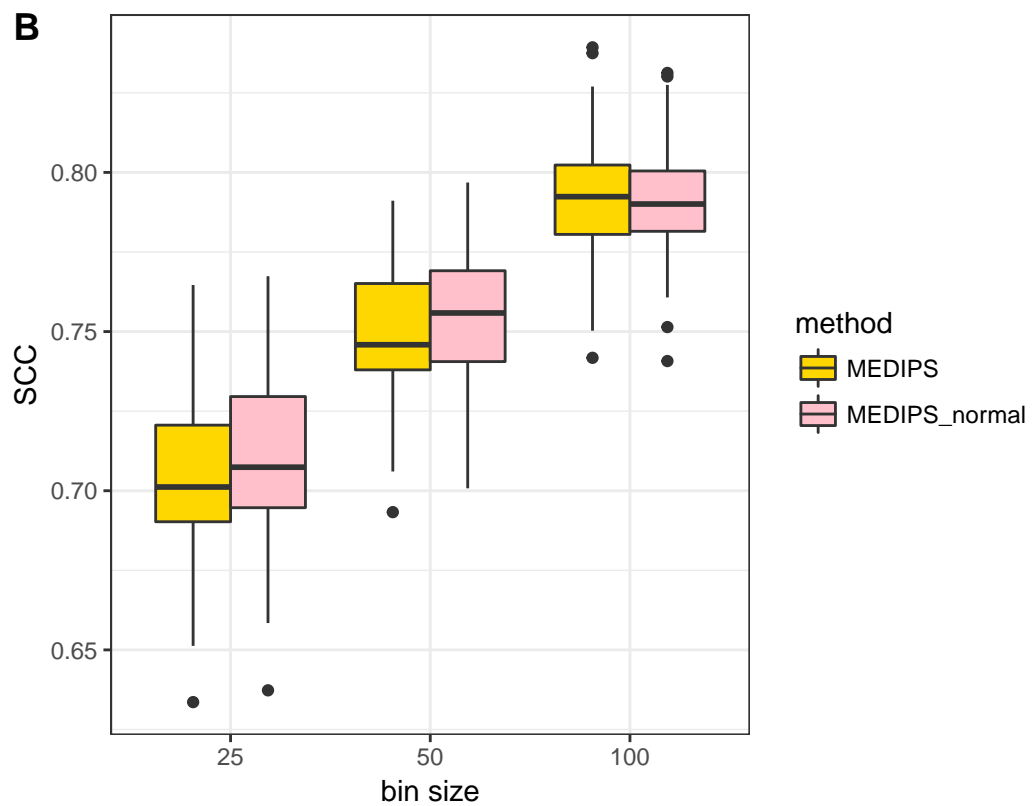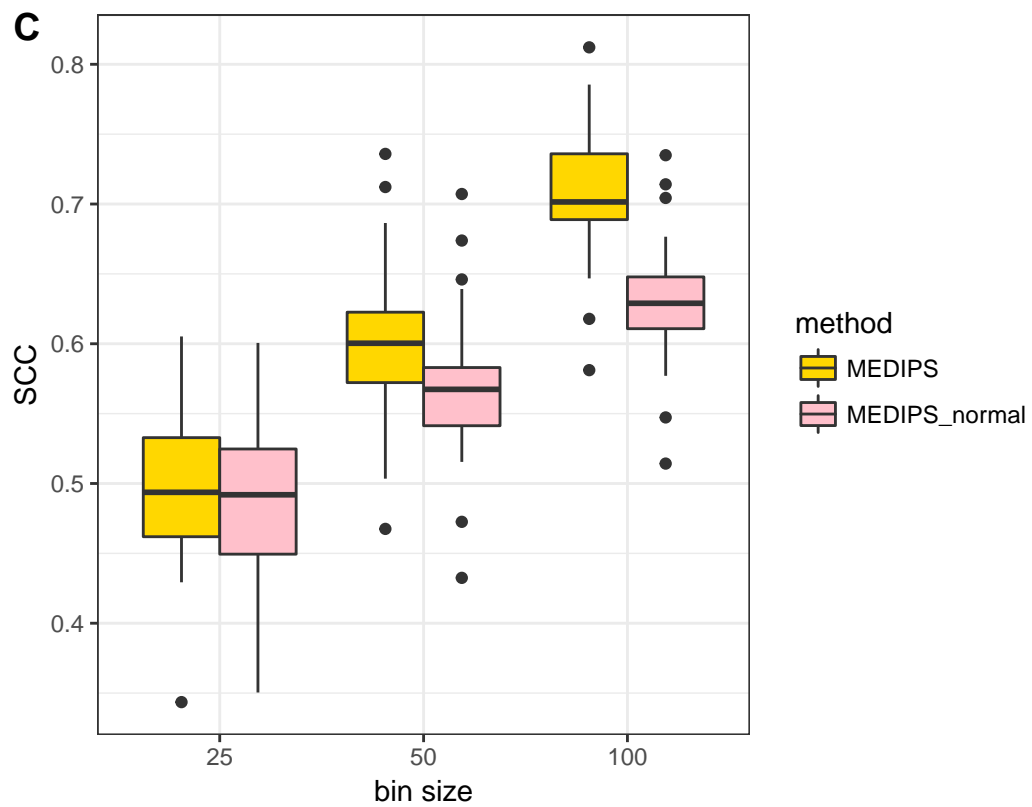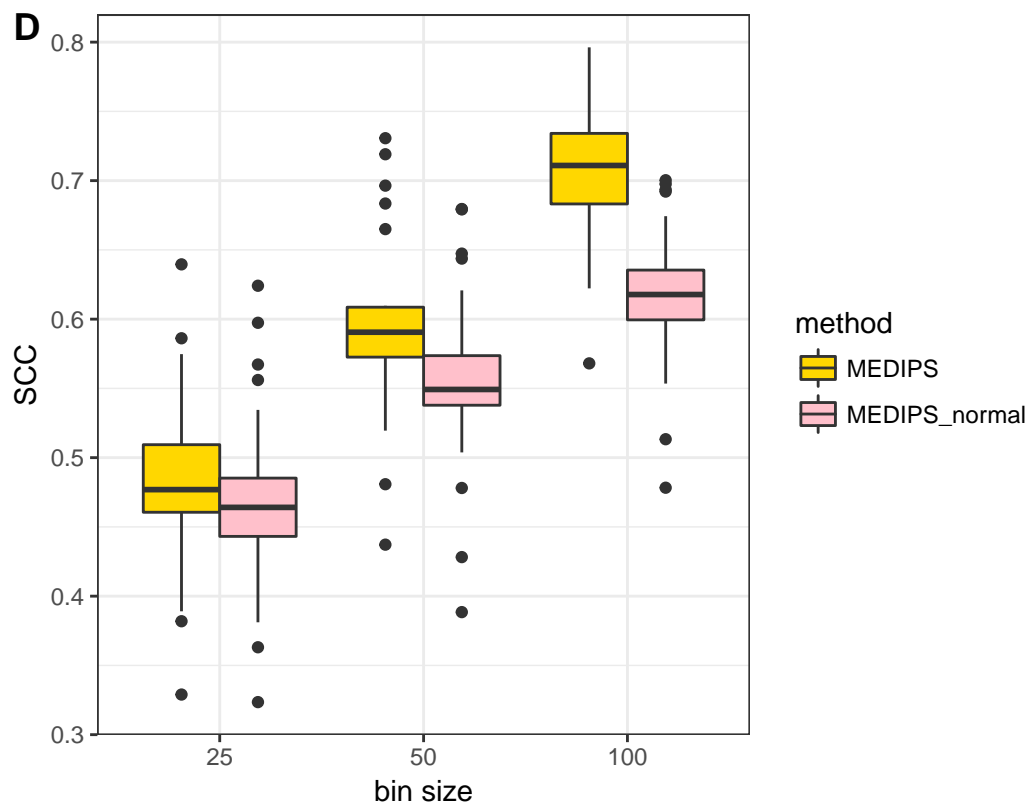

Supplement: Supplementary file 6 — Figure S2. Comparison of the methods MEDIPS_normal and MEDIPS. Spearman correlation coefficient (SCC) between MeDIP-seq and RRBS data calculated for four cell types. A GM12878; B K562; C foreskin fibroblasts; and D mammary epithelial cells. Y-axis shows the SCC values. X-axis shows the varying parameter bin size from 25 bp to 100 bp. Boxplot illustrates the variation of SCC across the 22 chromosomes. (PDF 10 kb) [file 12859_2018_2574_MOESM6_ESM.pdf]

**A**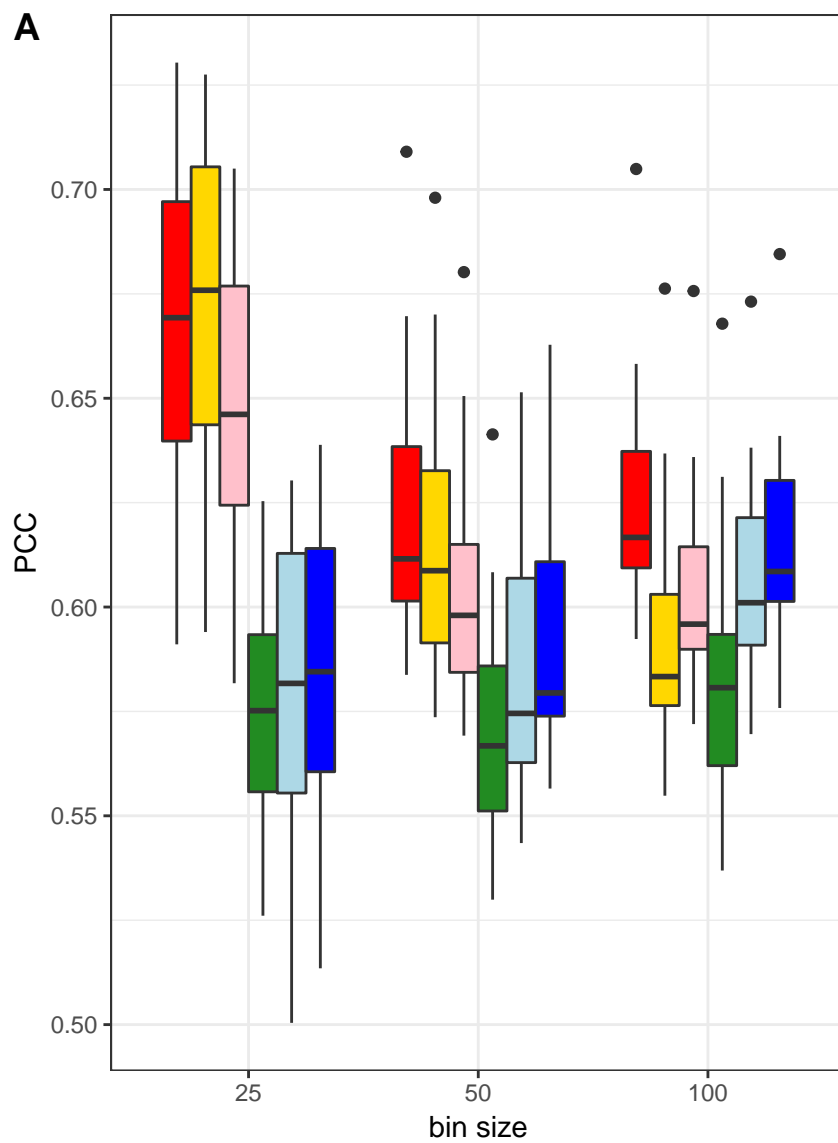**B**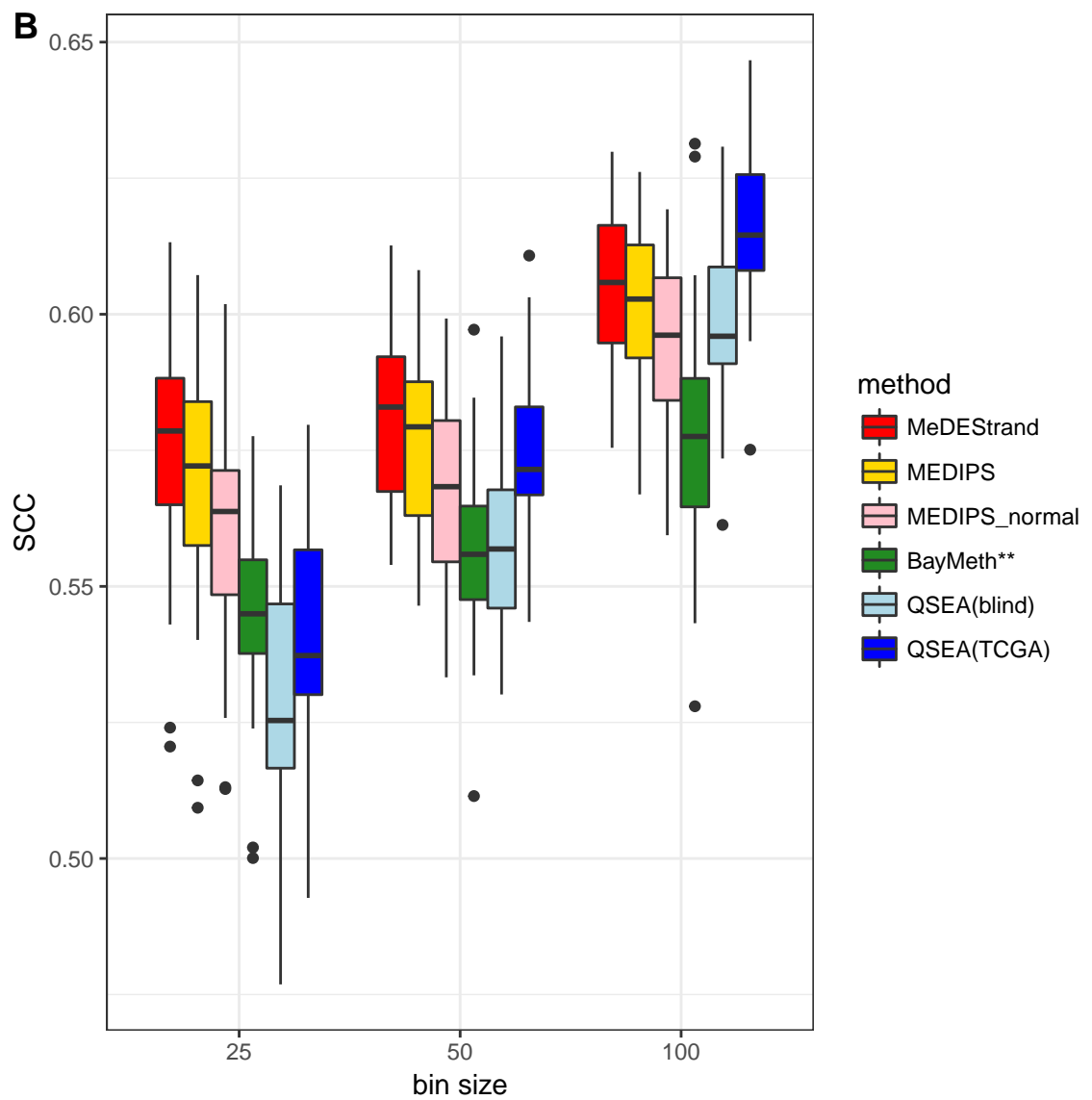

Supplement: Supplementary file 7 — Figure S3. Comparison of all methods for inferring CpG methylation levels based on DNA enrichment data. A Pearson (PCC) and B Spearman correlation coefficients (SCC) between the MeDIP-seq and WGBS data for the GM12878 cell line are calculated. Y-axis shows the PCC or SCC values. X-axis shows the varying parameter bin size from 25 bp to 100 bp. Boxplot illustrates the variation of PCC and SCC across the 22 chromosomes. (PDF 9 kb) [file 12859_2018_2574_MOESM7_ESM.pdf]
